# Central nervous system symptom burden and clinical implications for antiemetic management with olanzapine in AC chemotherapy

**DOI:** 10.1093/oncolo/oyag351

**Published:** 2026-09-09

**Authors:** Ryoko Udagawa, Mototsugu Shimokawa, Hirotoshi Iihara, Rie Ozeki, Michiko Tsuneizumi, Manabu Futamura, Tomoyuki Fujita, Hideaki Ogata, Hiroko Bando, Toshitaka Uomori, Hironobu Hashimoto, Ryosuke Hayami, Yoshihisa Tokumaru, Goro Kutomi, Kan Yonemori, Ryoichi Matsunuma, Mika Kitahora, Naotake Yanagisawa, Tatsunori Shimoi, Mitsue Saito

**Affiliations:** Department of Pharmacy, National Cancer Center Hospital, Chuo, Tokyo, 104-0045, Japan; Department of Biostatistics, Yamaguchi University Graduate School of Medicine, Ube, Yamaguchi, 755-8505, Japan; Department of Pharmacy, Gifu University Hospital, Gifu, Gifu, 501-1194, Japan; Cancer Center, Gifu University Hospital, Gifu, Gifu, 501-1194, Japan; Faculty of Pharmacy, Juntendo University, Urayasu, Chiba, 279-0013, Japan; Department of Breast Surgery, Shizuoka General Hospital, Shizuoka, Shizuoka, 420-8527, Japan; Department of Pharmacy, Gifu University Hospital, Gifu, Gifu, 501-1194, Japan; Department of Breast Surgery, Gifu University Hospital, Gifu, Gifu, 501-1194, Japan; Department of Breast Oncology, Juntendo Urayasu Hospital, Urayasu, Chiba, 279-0021, Japan; Department of Breast and Endocrine Surgery, Toho University Medical Center Omori Hospital, Ota, Tokyo, 143-8541, Japan; Department of Breast and Endocrine Surgery, Institute of Medicine, University of Tsukuba, Tsukuba, Ibaraki, 305-8575, Japan; Department of Breast Oncology, Faculty of Medicine, Juntendo University, Bunkyo, Tokyo, 113-8421, Japan; Department of Pharmacy, National Cancer Center Hospital, Chuo, Tokyo, 104-0045, Japan; Department of Breast Surgery, Shizuoka General Hospital, Shizuoka, Shizuoka, 420-8527, Japan; Department of Breast Surgery, Gifu University Hospital, Gifu, Gifu, 501-1194, Japan; Department of Breast Oncology, Faculty of Medicine, Juntendo University, Bunkyo, Tokyo, 113-8421, Japan; Department of Medical Oncology, National Cancer Center Hospital, Chuo, Tokyo, 104-0045, Japan; Department of Breast Surgery, Shizuoka General Hospital, Shizuoka, Shizuoka, 420-8527, Japan; Department of Pharmacy, Gifu University Hospital, Gifu, Gifu, 501-1194, Japan; Clinical Research and Trial Center, Juntendo University Hospital, Bunkyo, Tokyo, 113-8431, Japan; Department of Medical Oncology, National Cancer Center Hospital, Chuo, Tokyo, 104-0045, Japan; Department of Breast Oncology, Faculty of Medicine, Juntendo University, Bunkyo, Tokyo, 113-8421, Japan

**Keywords:** sedation, concentration impairment, CINV, olanzapine, anthracycline–cyclophosphamide chemotherapy, breast cancer

## Abstract

**Background:**

Olanzapine 5 mg improves chemotherapy-induced nausea and vomiting (CINV) control in patients receiving anthracycline–cyclophosphamide (AC) chemotherapy, but sedation remains a concern. We evaluated whether central nervous system (CNS) symptom burden identifies patients at higher risk of poor CINV control.

**Patients and methods:**

This secondary analysis of the phase III JTOP-B trial included women with breast cancer receiving AC chemotherapy. Patients were randomized to olanzapine 5 mg or placebo plus standard triplet antiemetic therapy. Daily patient-reported sedation and concentration impairment were assessed for 5 days. CNS burden was evaluated using recurrent “Bad Days” (concurrent moderate/severe sedation and concentration impairment) and cumulative CNS burden scores. Associations between CNS symptom patterns and complete response (CR; no emesis and no rescue medication) during 0-120 hours were analyzed.

**Results:**

Among 480 patients, CR rates decreased with increasing CNS symptom severity in both groups. In the olanzapine group, CR rates decreased across increasing sedation (75.0%-51.6%) and concentration impairment (78.1%-48.6%). Concentration impairment more strongly identified patients with poor CINV control than sedation alone. Higher cumulative CNS burden and recurrent Bad Days were associated with lower CR rates, identifying a clinically vulnerable population. Olanzapine maintained higher CR rates than placebo across CNS symptom strata.

**Conclusions:**

CNS symptom burden was associated with suboptimal CINV control, while 5 mg of olanzapine maintained superior antiemetic efficacy across CNS symptom strata. Monitoring day-to-day CNS symptoms may help identify patients requiring closer observation or additional supportive care.

**Trial registration:**

Japan Registry of Clinical Trials Identifier: jRCT1031200134

Implications for practice Central nervous system (CNS) symptoms during antiemetic prophylaxis may reflect a clinically vulnerable state associated with poor chemotherapy-induced nausea and vomiting control rather than olanzapine-related toxicity alone. Concentration impairment and co-occurring CNS symptoms identified patients with particularly poor antiemetic outcomes, while olanzapine maintained efficacy regardless of symptom severity. These findings support longitudinal monitoring of CNS symptoms to identify patients who may benefit from earlier supportive care interventions rather than discontinuation of olanzapine.

## Introduction

Chemotherapy-induced nausea and vomiting (CINV) remains a major barrier to treatment adherence and quality of life, even in the era of modern antiemetic prophylaxis. In the phase III JTOP-B trial (jRCT1031200134; https://jrct.mhlw.go.jp/en-latest-detail/jRCT1031200134),^1^ the addition of olanzapine 5 mg to standard triplet therapy significantly increased complete response (CR) rates during the 0-120-hour period for patients receiving anthracycline–cyclophosphamide (AC) chemotherapy, without an increase in serious adverse events,^2^ establishing olanzapine 5 mg as a practical and effective antiemetic option in outpatient settings.

Olanzapine’s broad receptor-binding profile includes antagonism at histamine H1 and muscarinic M1 receptors, pharmacologic actions well known to cause sedation and impair attention, particularly at higher doses.^3^^,^^4^ What remains uncertain, and highly relevant to daily oncology care, is how sedation and cognitive-related symptoms, particularly difficulties in attention or concentration, evolve after chemotherapy and whether their fluctuations bear any relationship to antiemetic efficacy.

A series of symptom clustering studies has shown that CINV seldom emerges in isolation but frequently co-occurs with fatigue, cognitive complaints, sleep disturbance, or psychological symptoms.^5–10^ Patients with prominent neuropsychological symptoms, including impaired concentration or high stress, often experience more severe or persistent CINV and higher gastrointestinal symptom burdens.^5–8^ Together with mechanistic evidence linking fatigue and cognitive dysfunction to shared pathways such as inflammation and altered neurotransmission,^11^ these findings suggest that central nervous system (CNS) symptoms during chemotherapy reflect not only adverse effects of supportive medications but also involvement in a broader neuropsychological symptom network intersecting with CINV. Chemotherapy-related cognitive changes have also been reported across diverse regimens, even in the absence of sedating agents, indicating potential cumulative cognitive vulnerability over treatment cycles.^12^

The directionality of these relationships remains unclear. Poorly controlled nausea may secondarily impair cognitive clarity through physiological or behavioral consequences, while excessive sedation or attentional lapses may hinder adequate self-care or timely use of rescue antiemetics, potentially predisposing patients to CINV breakthrough. Neuropsychological studies further suggest a bidirectional interplay between nausea and cognitive symptoms.^7^^,^^8^ However, no studies have examined day-to-day variations in sedation and cognitive-related symptoms after chemotherapy, nor whether distinct CNS states, such as sedation with preserved attention versus sedation accompanied by attentional or concentration difficulties, differentially relate to antiemetic effectiveness.

Against this background, this secondary analysis of the JTOP-B trial examined daily patterns of sedation and concentration impairment after AC chemotherapy and their relationship to antiemetic outcomes to identify clinically meaningful CNS symptom states relevant to CINV control.

## Patients and methods

### Study design: overview of the JTOP-B trial

The JTOP-B trial was a phase III, multicenter, randomized, double-blind, placebo-controlled trial conducted at 15 institutions across Japan. Between October 2020 and November 2022, 500 female patients with stage I-III primary breast cancer scheduled to receive their first cycle of AC-based chemotherapy were enrolled and randomly assigned in a 1:1 ratio to receive either olanzapine 5 mg or placebo, in addition to standard triplet antiemetic therapy with palonosetron, aprepitant, and dexamethasone.

Olanzapine was self-administered at home once on day 1, within 5 hours following chemotherapy and before dinner, and then once daily after dinner on days 2-4. As described in the primary report, this administration schedule was designed to synchronize peak plasma levels of olanzapine with patients’ sleep cycles, thereby minimizing the risk of sedation-related adverse events during outpatient care.^2^

The primary endpoint was the CR rate during the 0-120-hour period post-chemotherapy, defined as no vomiting and no rescue medication use. Secondary endpoints included CR during the acute (0-24 h), delayed (24-120 h), and extended delayed (24-168 h) phases, as well as the extended overall period (0-168 h). Additional endpoints included complete control (CC), total control (TC), time to treatment failure (TTF), adverse event profiles, and patient satisfaction with antiemetic therapy. Symptom diaries and validated patient-reported outcome tools, including the PRO-CTCAE, were used for evaluation.

In addition to nausea- and vomiting-related endpoints, patients completed daily paper-based diaries over a 7-day period to report the severity of specific symptoms including daytime somnolence and concentration impairment, using 4-point Likert-type scales (0: none, 1: mild, 2: moderate, 3: severe). These entries were reviewed as part of the broader tolerability and patient experience assessment.

The trial demonstrated that olanzapine significantly improved CR, CC, TC, and TTF compared with placebo, with a 22.7% absolute increase in CR during the overall phase. Daytime somnolence was more frequently reported in the olanzapine group, but it did not lead to a higher incidence of concentration impairment or treatment discontinuation. These findings support the safe and effective use of olanzapine 5 mg in outpatient AC-based chemotherapy settings for high-risk patients. ^2^

### Secondary analysis framework

Although this was a post hoc secondary analysis, the analytical framework focusing on daily sedation and concentration impairment and their composite measures was defined prior to the conduct of the present analyses. This secondary analysis evaluated daily patient-reported patterns of sedation and concentration impairment during the 5 days following AC chemotherapy in both the olanzapine and placebo groups. In this secondary analysis, the term “sedation” was used to refer to daytime somnolence as assessed in the original trial. The Full Analysis Set (FAS) was used for all analyses. Although symptom diaries were collected for 7 days, only data from days 1 through 5 were included, corresponding to the period used to define the primary endpoint (CR from 0-120 hours).

To characterize CNS symptom burden, 2 composite metrics were derived from daily sedation and concentration impairment scores (each 0-3). Bad Days were defined as days on which both sedation and concentration impairment scores were ≥2. The CNS Burden Score was calculated as the sum of daily sedation and concentration impairment scores across days 1 through 5 (range 0-30; maximum 6 points per day × 5 days). These metrics were analyzed in association with antiemetic efficacy outcomes (CR, CC, and TC) during the 0-120-hour period.

### Statistical analysis

All analyses were conducted using the FAS. Baseline characteristics and symptom scores were summarized using Descriptive statistics. The number and proportion of patients who achieved complete response, complete control, and total control were calculated within subgroups defined by each composite metric (eg, presence or absence of Bad Days, or by Burden Score tertiles).

Categorical variables were calculated as frequencies and proportions, and continuous variables, including the CNS Burden Score, were summarized using descriptive statistics. Between-group differences in continuous variables were assessed using the Mann–Whitney *U* test. The joint distribution of the Burden Score and cognitive impairment score in relation to complete response (CR) was evaluated using heatmaps.

All statistical tests were 2-sided, and a *P*-value of ≤.05 was considered statistically significant. Statistical analyses were performed using SAS software, version 9.4 (SAS Institute).

## Results

### Patient characteristics

**Figure 1.**
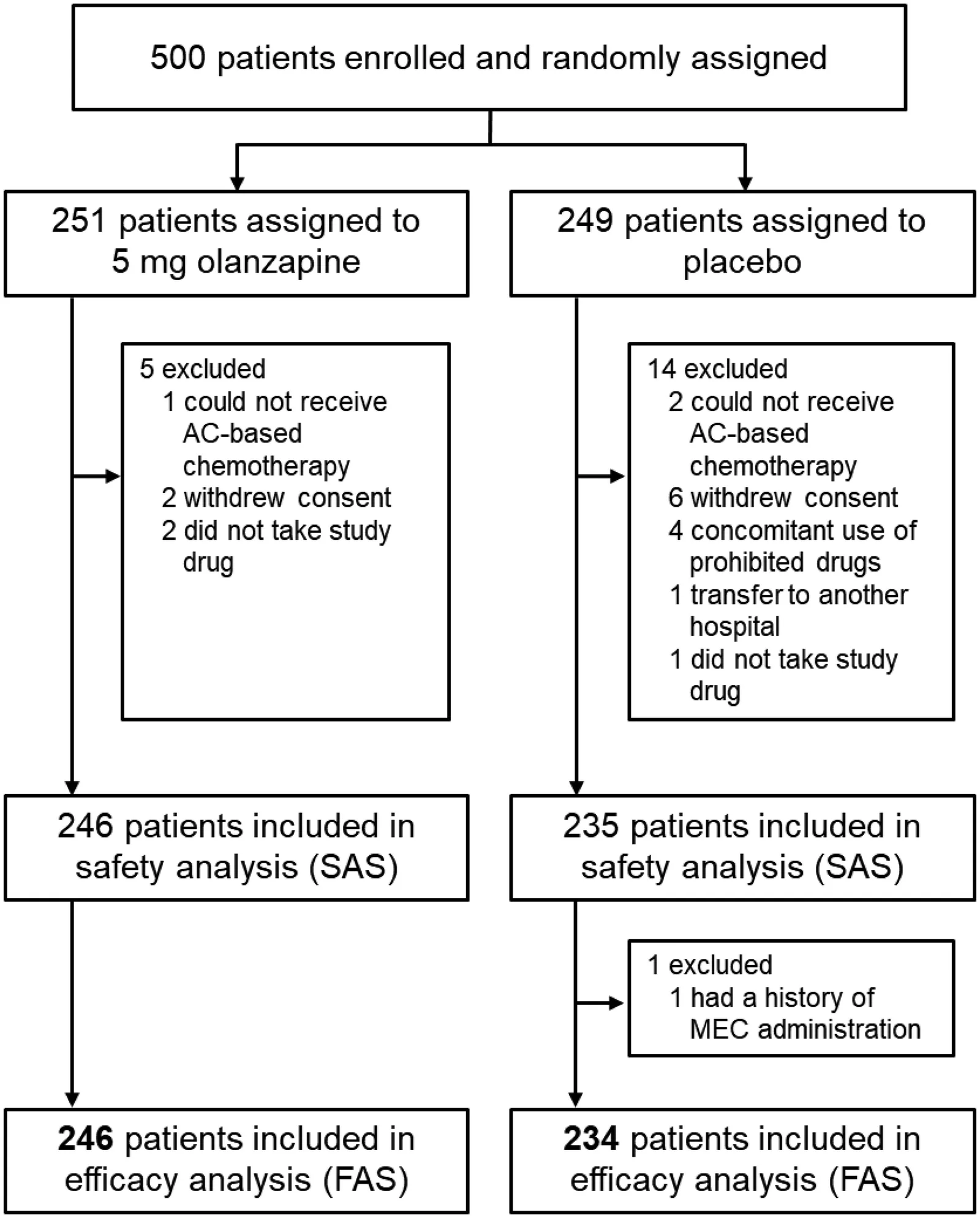
CONSORT diagram: Phase III JTOP-B trial. This secondary analysis included patients who completed symptom diaries for days 1 through 5 after chemotherapy (*n* = 480). AC, anthracycline–cyclophosphamide; FAS, full analysis set; MEC, moderately emetogenic chemotherapy; SAS, safety analysis set.

A total of 480 patients were included in the analysis (Figure 1). Baseline characteristics were well balanced between the olanzapine group (*n *= 246) and the placebo group (*n *= 234; Table 1). The median age was 52 years (IQR 45-60) in the olanzapine group and 51 years (46-60) in the placebo group. The proportion of patients aged <55 years was 59% in both groups. Eastern Cooperative Oncology Group performance status of 0 was reported in 99% of patients receiving olanzapine and 100% receiving placebo. Chemotherapy regimens were similarly distributed, with epirubicin plus cyclophosphamide (EC) being the most common (37% and 34%, respectively), followed by dose-dense EC (27% vs 32%) and doxorubicin plus cyclophosphamide (28% vs 25%).

**Table 1.** Patient characteristics and chemotherapy regimens.

		Olanzapine group (*n *= 246)	Placebo group (*n *= 234)
**Age, years (median [IQR])**		52 (45-60)	51 (46-60)
	<55	144 (58.5%)	138 (59.0%)
	≥55	102 (41.5%)	96 (41.0%)
**ECOG PS at baseline, *n* (%)**			
	0	244 (99.2%)	234 (100.0%)
	1	2 (0.8%)	0 (0.0%)
**Chemotherapy regimen, *n* (%)**			
	AC	68 (27.6%)	58 (24.8%)
	EC	90 (36.6%)	79 (33.8%)
	ddAC	13 (5.3%)	17 (7.3%)
	ddEC	67 (27.2%)	74 (31.6%)
	FEC	8 (3.3%)	6 (2.6%)
**Morning sickness, *n* (%)**			
	Yes	106 (43.1%)	113 (48.3%)
	No	89 (36.2%)	60 (25.6%)
	No pregnancy experience	51 (20.7%)	61 (26.1%)
**Motion sickness, *n* (%)**			
	Yes	70 (28.5%)	83 (35.5%)
	No	176 (71.5%)	151 (64.5%)
**Drinking habit, *n* (%)**			
	Do not drink	131 (53.3%)	109 (46.6%)
	≤Once a month	26 (10.6%)	45 (19.2%)
	2-4 times a month	24 (9.8%)	16 (6.8%)
	2-3 times a week	21 (8.5%)	32 (13.7%)
	≥4 times a week	44 (17.9%)	32 (13.7%)

Abbreviations: AC, doxorubicin + cyclophosphamide; ddAC, dose dense AC; ddEC, dose dense EC; EC, epirubicin + cyclophosphamide; ECOG PS, Eastern Cooperative Oncology Group performance status; FEC, 5-fluorouracil + epirubicin + cyclophosphamide; IQR, interquartile range.

Additional risk factors for chemotherapy-induced nausea and vomiting, such as motion sickness (28% vs 35%) and morning sickness during pregnancy (43% vs 48%), were also comparable between the 2 groups. Alcohol consumption patterns were balanced, though a higher proportion of patients in the olanzapine group reported drinking ≥4 times a week (18% vs 14%).

### Sedation and antiemetic outcomes

In the olanzapine group, CR, CC, and TC rates decreased progressively with increasing sedation severity. CR declined from 75.0% in patients without sedation to 63.3%, 56.8%, and 51.6% in those with mild, moderate, and severe sedation, respectively. CC decreased from 75.0% to 46.9%, and TC from 62.5% to 29.7% across the same categories (Table 2).

**Table 2.** Antiemetic outcomes according to sedation severity.

Outcomes *n* (%)	Olanzapine (*n *= 246)				Placebo (*n *= 234)			
	0: None (*n *= 8)	1: Mild (*n *= 79)	2: Moderate (*n *= 95)	3: Severe (*n *= 64)	0: None (*n *= 12)	1: Mild (*n *= 100)	2: Moderate (*n *= 84)	3: Severe (*n *= 38)
**CR**	6 (75.0)	50 (63.3)	54 (56.8)	33 (51.6)	3 (25.0)	43 (43.0)	29 (34.5)	8 (21.1)
**CC**	6 (75.0)	50 (63.3)	53 (55.8)	30 (46.9)	3 (25.0)	41 (41.0)	23 (27.4)	8 (21.1)
**TC**	5 (62.5)	35 (44.3)	34 (35.8)	19 (29.7)	2 (16.7)	29 (29.0)	12 (14.3)	3 (7.9)

Abbreviations: CC, complete control; CR, complete response; TC, total control.

In the placebo group, response rates were lower overall and tended to be lower in patients with higher sedation scores, although the pattern was not strictly monotonic across categories, with CR ranging from 25.0% to 21.1%, CC from 25.0% to 21.1%, and TC from 16.7% to 7.9% (Table 2). Given the small sample size in the least-sedated category, these estimates should be interpreted cautiously. Across all sedation strata, response rates consistently favored olanzapine over placebo.

### Concentration impairment and antiemetic outcomes

In the olanzapine group, CR, CC, and TC rates also decreased progressively with increasing concentration impairment. CR declined from 78.1% in patients without impairment to 60.5%, 49.2%, and 48.6% in those with mild, moderate, and severe impairment, respectively. CC and TC showed parallel decreases across the same categories (Table 3). In the placebo group, response rates were lower overall and tended to be lower in patients with higher cognitive impairment scores, although the pattern was not strictly monotonic across categories, with CR ranging from 38.5% to 13.8%, CC from 35.9% to 13.8%, and TC from 28.2% to 3.4% (Table 3). As with sedation, sample sizes in several concentration impairment categories were small, and estimates should be interpreted cautiously. Across all concentration impairment strata, response rates again favored olanzapine over placebo.

**Table 3.** Antiemetic outcomes according to concentration impairment severity.

Outcomes *n* (%)	Olanzapine (*n *= 246)				Placebo (*n *= 234)			
	0: None (*n *= 32)	1: Mild (*n *= 114)	2: Moderate (*n *= 65)	3: Severe (*n *= 35)	0: None (*n *= 39)	1: Mild (*n *= 102)	2: Moderate (*n *= 64)	3: Severe (*n *= 29)
**CR**	25 (78.1)	69 (60.5)	32 (49.2)	17 (48.6)	15 (38.5)	43 (42.2)	21 (32.8)	4 (13.8)
**CC**	25 (78.1)	67 (58.8)	31 (47.7)	16 (45.7)	14 (35.9)	40 (39.2)	17 (26.6)	4 (13.8)
**TC**	20 (62.5)	40 (35.1)	23 (35.4)	10 (28.6)	11 (28.2)	26 (25.5)	8 (12.5)	1 (3.4)

Abbreviations: CC, complete control; CR, complete response; TC, total control.

### Joint distribution of sedation and concentration impairment

**Figure 2.**
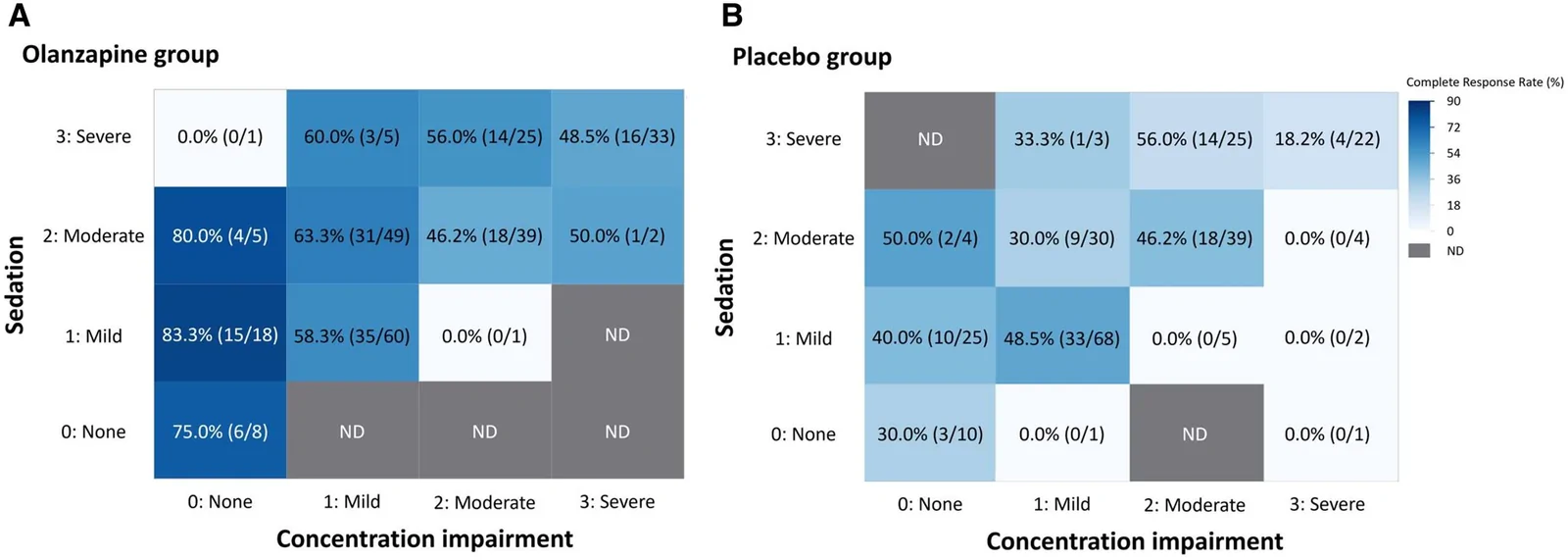
Joint heatmap of sedation and concentration impairment by complete response. Heatmaps showing complete response (CR) rates according to the combined severity of sedation and concentration impairment in the olanzapine group (A) and the placebo group (B). Sedation severity is shown on the y-axis and concentration impairment on the x-axis, each categorized as none (0), mild (1), moderate (2), or severe (3). Cell colors represent CR rates (%), with darker shades indicating higher response rates. Exact percentages and the number of patients achieving CR over the total number in each category (*n*/*N*) are shown within each cell. Gray cells labeled “ND” indicate categories in which no patients were available for analysis. ND, not determined.

In the olanzapine group, the joint distribution of sedation and concentration impairment showed a consistent pattern across the heatmap. Patients with low concentration impairment generally maintained higher CR rates across most sedation categories, whereas those with higher concentration impairment scores exhibited lower CR values even when sedation was minimal (Figure 2A). Several strata contained no patients and were therefore not estimable.

The placebo group showed a similar overall pattern, with lower absolute CR values across nearly all combinations of sedation and concentration impairment scores (Figure 2B). As in the olanzapine arm, some strata had no patients, and corresponding CR values could not be calculated.

### Burden score and antiemetic outcomes

**Figure 3.**
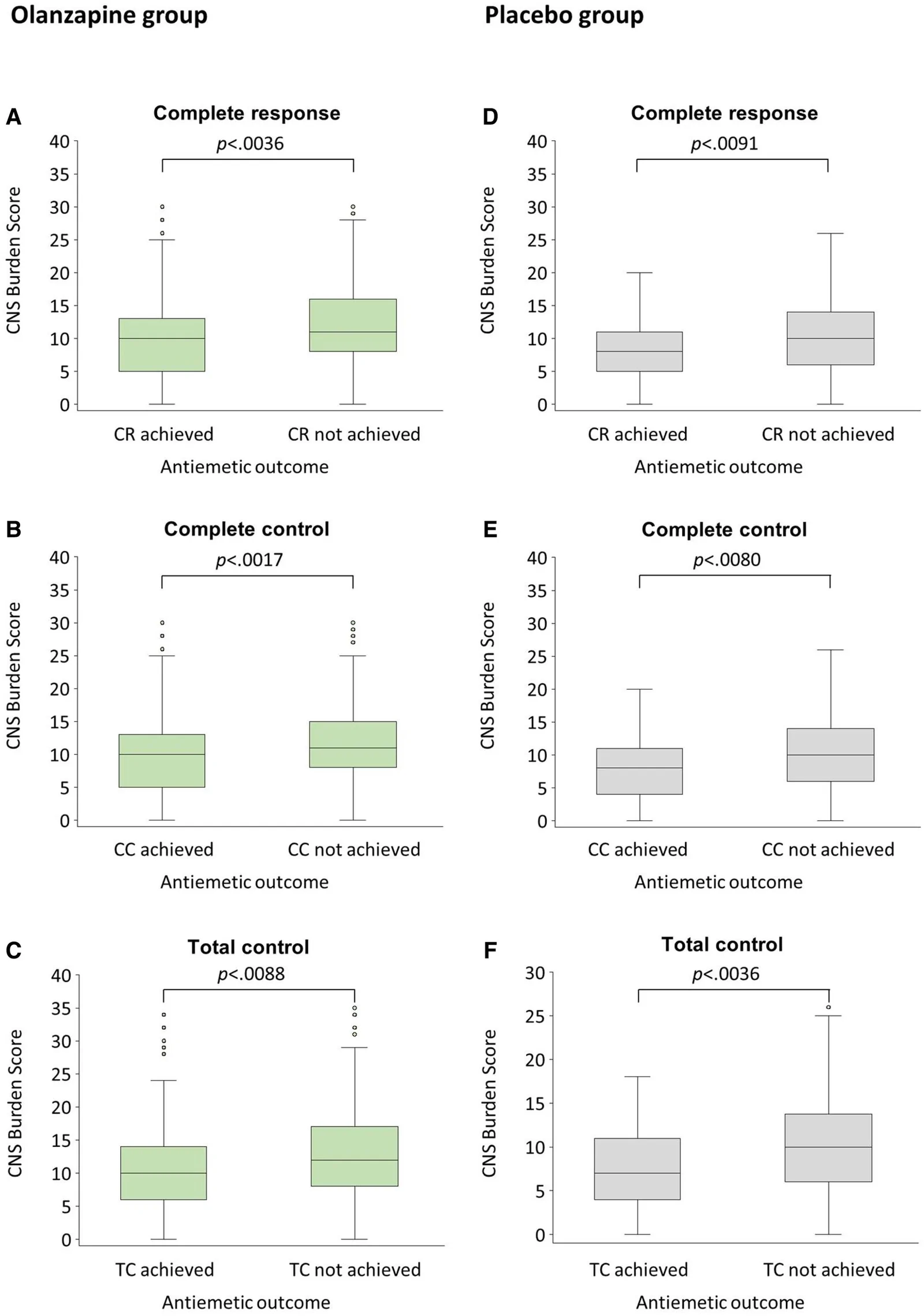
CNS Burden Score according to antiemetic outcomes in the olanzapine and placebo groups. Box-and-whisker plots show the distribution of CNS Burden Scores according to antiemetic outcomes in the olanzapine group (A-C) and the placebo group (D-F). For each outcome (complete response [CR], complete control [CC], and total control [TC]), patients are stratified by whether the outcome was achieved or not achieved. Boxes represent the interquartile range (IQR), with the horizontal line indicating the median. Whiskers extend to 1.5 times the IQR, and individual points indicate outliers. CNS, central nervous system.

In the olanzapine group, patients who achieved CR had lower CNS Burden Scores than those who did not. Median Burden Scores were 10 in the CR group and 11 in the non-CR group, with a visible upward shift in the overall distribution among non-CR patients (*P* < .0036; Figure 3A). Similar patterns were observed for CC (median 10 vs 11; *P* < .0017; Figure 3B) and TC (median 9 vs 11; *P* < .0088; Figure 3C). Although the distributions overlapped substantially, higher Burden Scores tended to be associated with lower rates of CR, CC, and TC.

A comparable trend was observed in the placebo group. Median Burden Scores were lower in patients who achieved CR compared with those who did not (8 vs 10; *P* < .0091; Figure 3D), with corresponding distributional shifts toward higher values in non-CR patients. Similar differences were seen for CC (median 8 vs 10; *P* < .0080; Figure 3E) and TC (median 7 vs 10; *P* < .0036; Figure 3F). As in the olanzapine group, patients with higher Burden Scores tended to have poorer antiemetic outcomes, despite considerable overlap between groups.

### Bad Days and antiemetic outcomes

In the olanzapine group, CR rates decreased across higher Bad-Day categories, from 63.5% in patients with no Bad Days to 59.5% with 1 day and 48.0% with 2 days. CR was 61.5% among those with 3 days, although this estimate was based on a small number of patients, and 27.3% and 33.3% among those with 4 and 5 days, respectively (Table 4).

**Table 4.** Antiemetic outcomes according to the number of bad days.

Outcomes *n* (%)	Olanzapine (*n *= 246)						Placebo (*n *= 234)					
	0 days (*n *= 148)	1 day (*n *= 37)	2 days (*n *= 25)	3 days (*n *= 13)	4 days (*n *= 11)	5 days (*n *= 12)	0 days (*n *= 149)	1 day (*n *= 29)	2 days (*n *= 22)	3 days (*n *= 24)	4 days (*n *= 8)	5 days (*n *= 2)
**CR**	94 (63.5)	22 (59.5)	12 (48.0)	8 (61.5)	3 (27.3)	4 (33.3)	58 (38.9)	10 (34.5)	6 (27.3)	7 (29.2)	2 (25.0)	0 (0.0)
**CC**	92 (62.2)	21 (56.8)	12 (48.0)	7 (53.8)	3 (27.3)	4 (33.3)	54 (36.2)	9 (31.0)	5 (22.7)	6 (25.0)	1 (12.5)	0 (0.0)
**TC**	60 (40.5)	16 (43.2)	8 (32.0)	4 (30.8)	2 (18.2)	3 (25.0)	37 (24.8)	3 (10.3)	4 (18.2)	2 (8.3)	0 (0.0)	0 (0.0)

Abbreviations: CC, complete control; CR, complete response; TC, total control.

In the placebo group, CR decreased from 38.9% in patients with no Bad Days to values ranging from 0% to 29.2% across higher Bad-Day categories with 3-5 days (Table 4). Across both treatment arms, higher numbers of Bad Days corresponded to lower CR estimates, although several strata contained few patients and should be interpreted cautiously.

## Discussion

In this secondary analysis of the phase III JTOP-B trial,^1^^,^^2^ patient-reported CNS symptoms, assessed as sedation and concentration impairment, were consistently associated with CINV outcomes in women receiving AC-based chemotherapy with or without olanzapine 5 mg. Across both arms, greater CNS symptom burden was associated with lower CR, CC, and TC rates, with similar downward trends observed in the placebo arm, supporting an underlying CNS vulnerability and symptom-cluster effect rather than drug-specific toxicity.^5–8^

Absolute response rates remained higher with olanzapine across all CNS symptom strata, indicating robust antiemetic efficacy despite the presence of CNS symptoms. Joint analyses further demonstrated that concentration impairment, more than sedation alone, discriminated patients with poorer CINV control. Patients experiencing recurrent Bad Days or higher cumulative CNS Burden Scores showed consistently lower CR, CC, and TC rates. Together, these findings suggest that overlapping and persistent CNS symptom burden, rather than sedation in isolation, characterizes a clinically vulnerable state in which antiemetic prophylaxis is less likely to succeed.

Several plausible mechanisms may underlie these observations. Poorly controlled CINV may exacerbate fatigue and cognitive dysfunction through physiological and behavioral consequences, while concurrent sedation and impaired concentration can hinder effective self-management, such as maintaining hydration, nutrition, or responding to early nausea. Together, these processes provide a clinically intuitive pathway through which CNS symptom burden may contribute to CINV breakthrough despite guideline-consistent prophylaxis. The higher CNS Burden Scores observed among non-responders are compatible with a bidirectional relationship between CNS symptoms and CINV control, rather than a 1-way effect of olanzapine-related sedation alone.^5–8^^,^^11^^,^^12^

Recent longitudinal data from the RxPONDER trial demonstrate that chemotherapy exposure is associated with persistent patient-reported cognitive impairment independent of menopausal status. While those findings address long-term cognitive effects over months to years, the present results extend this concept to the immediate post-chemotherapy period, showing that transient, day-to-day CNS vulnerability within the first treatment cycle is closely linked to CINV control.^13^

These results are consistent with the broader symptom cluster literature in oncology, in which nausea and vomiting commonly co-occur with fatigue, sleep disturbance, psychological distress, and cognitive complaints, and patients with prominent neuropsychological symptoms tend to experience more severe or persistent nausea and higher gastrointestinal symptom burdens.^5–8^

Importantly, the present analysis adds novel elements not previously described in randomized olanzapine trials.^2–4^ Sedation and concentration impairment were evaluated as distinct yet interacting dimensions, with concentration impairment emerging as a more discriminating marker of poor CINV control than sedation alone. By leveraging day-level data, we further introduced 2 complementary metrics—Bad Days and the CNS Burden Score—that capture episodic and cumulative CNS symptom burden over the 5-day risk window. Together, these results provide quantitative support for a familiar clinical distinction: patients with sedation but preserved attention differ meaningfully from those with sedation accompanied by concentration difficulties, with distinct implications for antiemetic effectiveness. The issue is not whether to discontinue olanzapine, but how to identify patients who may require additional supportive care. Monitoring concentration impairment, recurrent Bad Days, and cumulative CNS burden may help identify patients who remain at risk of poor CINV control.

Several limitations warrant consideration. The analyses were post hoc and exploratory, and the original trial was not powered to detect differences in CNS-derived metrics. Some heatmap cells and Bad-Day categories contained small numbers of patients, which led to unstable estimates and contributed to the irregular gradients seen at the extremes of symptom burden. Sedation and concentration impairment were assessed once per day using brief self-report scales, which may miss intra-day fluctuations and cannot fully distinguish between perceived somnolence, physical fatigue, and objective cognitive dysfunction. The study population consisted of Japanese women with early-stage breast cancer receiving AC-based chemotherapy and 5 mg of olanzapine; therefore, the generalizability of these findings to other populations, regimens, or dosing strategies may be limited and warrants further validation. Residual confounding by unmeasured baseline factors such as anxiety, depression, sleep disorders, prior cognitive difficulties, or concomitant medications is also possible. Finally, although the JTOP-B trial itself was a randomized controlled study, this exploratory post hoc analysis is observational, and therefore cannot determine causality between CNS symptom burden and CINV control.

## Conclusion

In this secondary analysis of the JTOP-B trial, patient-reported profiles of sedation and concentration impairment were associated with the likelihood of achieving adequate CINV control. Concentration impairment, the recurrence of “Bad Days,” and higher CNS Burden Scores consistently identified patients at risk for suboptimal outcomes. Notably, 5 mg of olanzapine maintained superior antiemetic efficacy across all CNS symptom strata, supporting its robustness even among patients experiencing CNS effects. This approach complements existing antiemetic strategies.

These findings support the clinical relevance of monitoring day-to-day CNS symptoms as part of routine supportive care. Incorporating CNS symptom patterns into risk assessment may help identify patients who require closer observation or adjustment of supportive strategies. Prospective studies are warranted to validate these symptom-derived metrics and to clarify their role in guiding individualized antiemetic management.

## Data Availability

The data generated in the JTOP-B trial and used in this study are available from the corresponding authors upon reasonable request, in accordance with the data-sharing policy of the original trial. Access to the data is subject to ethical and legal restrictions.
